# *MtPIN1* and *MtPIN3* Play Dual Roles in Regulation of Shade Avoidance Response under Different Environments in *Medicago truncatula*

**DOI:** 10.3390/ijms21228742

**Published:** 2020-11-19

**Authors:** Xue Zhang, Lu Liu, Hongfeng Wang, Zhiqun Gu, Yafei Liu, Minmin Wang, Min Wang, Yiteng Xu, Qingbiao Shi, Gang Li, Jianhua Tong, Langtao Xiao, Zeng-Yu Wang, Kirankumar S. Mysore, Jiangqi Wen, Chuanen Zhou

**Affiliations:** 1 The Key Laboratory of Plant Development and Environmental Adaptation Biology, Ministry of Education, School of Life Science, Shandong University, Qingdao 266237, China; zxue@mail.sdu.edu.cn (X.Z.); liulu9509@163.com (L.L.); zqgu@mail.sdu.edu.cn (Z.G.); liuyafei199508@163.com (Y.L.); minminw@mail.sdu.edu.cn (M.W.); 201820281@mail.sdu.edu.cn (M.W.); 201411612@mail.sdu.edu.cn (Y.X.); 2 School of Life Science, Guangzhou University, Guangzhou 510006, China; wanghf@gzhu.edu.cn; 3 State Key Laboratory of Crop Biology, College of Life Sciences, Shandong Agricultural University, Tai’an 271018, China; sqb900918@163.com (Q.S.); gangli@sdau.edu.cn (G.L.); 4 Hunan Provincial Key Laboratory of Phytohormones, Hunan Agricultural University, Changsha 410128, China; tjh0421@sohu.com (J.T.); langtaoxiao@163.com (L.X.); 5 Grassland Agri-Husbandry Research Center, Qingdao Agricultural University, Qingdao 266109, China; zywang@qau.edu.cn; 6 Noble Research Institute, LLC, Ardmore, OK 73401, USA; ksmysore@noble.org (K.S.M.); jwen@noble.org (J.W.)

**Keywords:** auxin, *Medicago truncatula*, PIN-FORMED, shadeavoidance response

## Abstract

Polar auxin transport mediated by PIN-FORMED (PIN) proteins is critical for plant growth and development. As an environmental cue, shade stimulates hypocotyls, petiole, and stem elongation by inducing auxin synthesis and asymmetric distributions, which is modulated by PIN3,4,7 in *Arabidopsis*. Here, we characterize the *MtPIN1* and *MtPIN3*, which are the orthologs of *PIN3*,*4*,*7*, in model legume species *Medicago truncatula*. Under the low Red:Far-Red (R:FR) ratio light, the expression of *MtPIN1* and *MtPIN3* is induced, and shadeavoidance response is disrupted in *mtpin1 mtpin3* double mutant, indicating that *MtPIN1* and *MtPIN3* have a conserved function in shade response. Surprisingly, under the normal growth condition, *mtpin1 mtpin3* displayed the constitutive shade avoidance responses, such as the elongated petiole, smaller leaf, and increased auxin and chlorophyll content. Therefore, *MtPIN1* and *MtPIN3* play dual roles in regulation of shadeavoidance response under different environments. Furthermore, these data suggest that *PIN3*,*4*,*7* and its orthologs have evolved conserved and specific functions among species.

## 1. Introduction

As a key plant hormone, auxin regulates diverse aspects of plant growth and developmental processes. At the organ levels, auxin is involved in the establishment and maintenance of apical dominance, phototropism, and gravitropism. At the cellular levels, auxin plays a prominent role in regulating cell division and cellular expansion by altering cell wall plastics [1]. Meanwhile, auxin is also a key regulator in regulating shade avoidance [2,3,4,5].

Indole-3-acetic acid (IAA) is the main natural auxin in plants and its biosynthesis mainly depends on two ways: de novo auxin biosynthesis and the release from auxin conjugates [6,7,8]. Auxin is mostly synthesized in young shoot tips and young leaves, and then transported to recipient organs for modulating diverse developmental processes. Two distinct but interconnected transport systems are involved in auxin transportation, one of them relies on phloem, which is fast but non-directional, and another manner is cell-to-cell polar auxin transportation (PAT), which is relatively slow but directional and precise [9]. PAT is mediated by three types of proteins: AUX1/LAX family [10], ABCB transporters [11,12,13] and PIN-FORMED (PIN) family of auxin efflux proteins [9,14]. PIN family auxin efflux carriers regulate PAT by altering their location at the plasma membrane to control the direction and quantity of auxin transportation [9,14].

Until now, PIN family proteins with eight members have been identified in *Arabidopsis*, namely, from PIN1 to PIN8 [14,15]. As previously reported, loss of function of PINs lead to diverse developmental defects in *Arabidopsis*. *pin1* is characterized with naked or pin-formed inflorescences, abnormal leaf, and loss of apical dominancy [16]. Besides, *pin1* also has defects in organ initiation and phyllotaxy formation [17,18]. PIN1 can generate auxin maximum in the outmost layer of meristem by directing auxin efflux and further controlling the establishment of leaf shape and margin [18,19,20]. Among eight PIN proteins in *Arabidopsis*, *PIN1*,*3*,*4*, and *7* are expressed in embryo. The developmental defects in early embryogenesis are found in *pin4* or *pin7* single mutants and in multiple mutant combinations [21]. While, *PIN3* and *PIN4* are specifically expressed in the outer side of the apical hook by increasing auxin drainage [22,23]. Similar to PIN1,4, and 7, PIN3 is localized at plasma membrane (PM) in a polar manner and can alter its subcellular localization during light and gravity stimuli [9]. *PIN3* is expressed in endodermis cells in dark-grown hypocotyls and distributed in a polar manner. Unilateral light and gravity stimulate PIN3 polarization to direct auxin flow toward a single side of the organ, resulting in hypocotyls’ differential growth [9,24,25]. In *M. truncatula*, eleven genes encoding PIN proteins have been identified [26,27]. Expression patterns analysis showed that the eleven *MtPINs* were differentially expressed in different tissues and organs [28]. Furthermore, a detailed expression analysis of *MtPIN* genes in *M. truncatula* root tips and nodules showed that *MtPIN9* is the only gene with a higher expression level in nodules than in roots [27]. Loss of function in *MtPIN2* led to disruption of basipetal auxin transport. However, *mtpin2* mutant could form nodules normally. Inoculation of wild-type roots increased the number of lateral roots. However, the number of lateral roots in *mtpin2* after inoculation was reduced [29]. Mutation in *SLM1/MtPIN10* resulted in pleiotropic phenotypes in various tissues, including cotyledon, leaf, and flower. In the *slm1/mtpin10* mutant, the distribution of auxin was disordered and compound leaf pattern was changed, which usually exhibited an increase in the number of terminal leaflets and a decrease in the number of lateral leaflets [30].

Plants selectively absorb red light in dense canopies and reflect far-red light formed by overtopping adjacent leaves. Hence, a sharp decline of the R:FR ratio is derived in a canopy [31,32,33,34]. For plants that are sessile, canopy shade derived from their neighbors limits the plants’ access to light for photosynthesis. Shaded plants struggle to escape from neighboring vegetation and develop a series of strategies called ‘shadeavoidance syndrome’ (SAS) [3,33,34,35,36,37]. In general, classical SAS phenotypes include increased growth of hypocotyls, petiole, internode, or stem, leaf hyponasty, decreased leaf lamina area, apical dominance, and early flowering time [31,32,33,35,38]. Increased growth of stem, leaf hyponasty, and apical dominance imply high auxin levels in these organs [33]. In *Arabidopsis*, low R:FR ratio light rapidly increases auxin levels by de novo auxin biosynthesis in cotyledons [39], and then the auxin is transported to hypocotyls [3]. As previously reported, loss of function of *PIN3* mutant displays no response to low R:FR, while low R:FR ratio light induces the expression and lateral distribution of PIN3 in hypocotyl, indicating that PIN3-mediated auxin redistribution is important in shadeavoidance response [24].

In this study, we reported the identification and characterization of *mtpin1* and *mtpin3* by screening *Tnt1* retrotransposon-tagged lines of *M. truncatula*. We found that mutations in *MtPIN1* and *MtPIN3* caused significant developmental defects due to the auxin accumulation in leaves and petioles. Both the reduced leaf area and elongated petiole of the double mutant suggested that *MtPIN1* and *MtPIN3* were involved in shadeavoidance response. Further analysis revealed that the expressions of *MtPIN1* and *MtPIN3* were induced by shade, and the double mutant failed to respond to low R:FR ratio light. These data illustrated that *MtPIN1* and *MtPIN3* play dual roles in regulating shadeavoidance response in *M. truncatula*.

## 2. Results and Discussion

### 2.1. Phylogenetic and Expression Pattern Analysis of MtPIN1 and MtPIN3

It has been reported that *mtpin10*/*slm1* exhibited striking defects in lateral organs in *M. truncatula* [30]. Among eleven MtPINs in *M. truncatula*, MtPIN1 and MtPIN3 were clustered together and close to the subclade of MtPIN10/MtPIN4/MtPIN5. Moreover, MtPIN1 and MtPIN3 were close to the subclade of *Arabidopsis* PIN3/PIN4/PIN7 (Supplementary Figure S1A) [26,27]. Both *MtPIN1* and *MtPIN3* were expressed in different organs and tissues including leaf, petiole, flower, root, and nodules, implying they play important roles in different developmental processes [27,28]. To better understand the possible functions of *MtPIN1* and *MtPIN3* in regulating organ development, we measured their expression patterns in different organs and tissues. Quantitative Real-time PCR (qRT-PCR) analysis showed that *MtPIN1* was highly expressed in petiole, juvenile leaf, and flowers, and expressed lower in shoot buds, stem, and seed (Supplementary Figure S1B). Similar to *MtPIN1*, *MtPIN3* was highly expressed in petiole and leaf (juvenile and adult leaf), and expressed relatively lower in other organs (Supplementary Figure S1C). The expression patterns of *MtPIN1* and *MtPIN3* indicated that they probably play an important role in modulating the development of petiole and leaf.

### 2.2. Isolation and Identification of Mutants of MtPIN1 and MtPIN3

**Figure 1:**
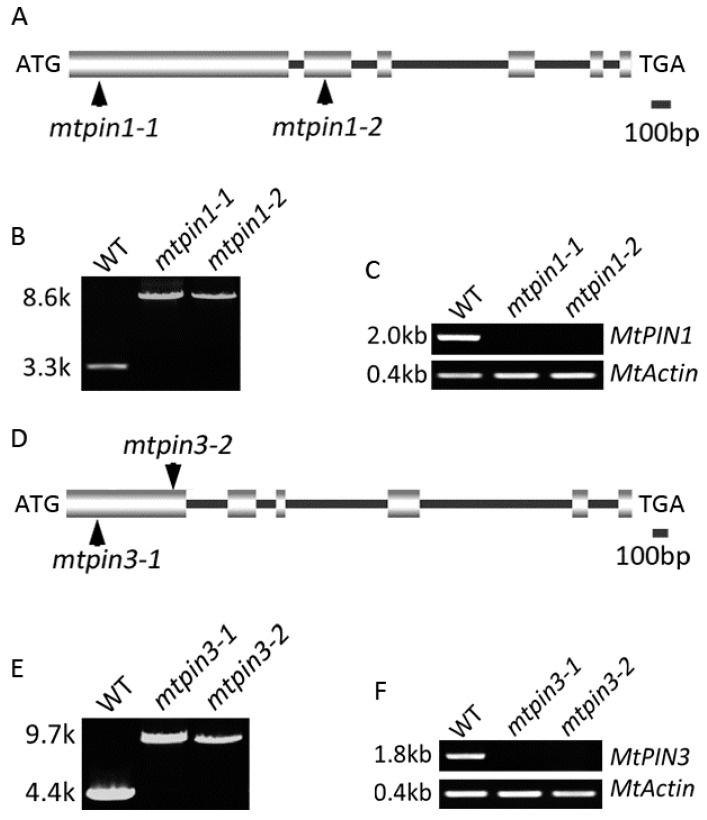
Molecular characterization of *MtPIN1* and *MtPIN3* in *M. truncatula*. (**A**,**D**) Schematic representation of the gene structure of *MtPIN1* and *MtPIN3*. The position of the ATG start and TGA stop codon are shown. Vertical arrows mark the *Tnt1* insertion site in *mtpin1* and *mtpin3*. (**B**,**E**) PCR amplification of *MtPIN1* and *MtPIN3* from wild-type (WT) and *mtpin1*, *mtpin3* mutants. A single *Tnt1* insertion (~5.3 kb) was detected in *mtpin1-1*, *mtpin1-2*, *mtpin3-1*, and *mtpin3-2*. (**C**,**F**) RT-PCR amplification of *MtPIN1* and *MtPIN3* transcripts in wild-type and mutants. *MtPIN1* and *MtPIN3* were not detected in *mtpin1-1*, *mtpin1-2*, *mtpin3-1*, and *mtpin3-2*. Actin was used as a loading control.

To further characterize *MtPIN1* and *MtPIN3*, firstly, their sequences were analyzed. Genome sequences and coding region sequences (CDS) of *MtPIN1* and *MtPIN3* were obtained from Phytozome (https://phytozome.jgi.doe.gov/). The full-length genomic sequence of *MtPIN1* was 3352 bp and CDS was 1980 bp. Alignment between the cDNA and genomic sequences of *MtPIN1* showed that *MtPIN1* consisted of 6 exons and 5 introns (Figure 1A). Similar to *MtPIN1*, *MtPIN3* contained 6 exons and 5 introns (Figure 1D). We further predicted the transmembrane domain of MtPIN1 and MtPIN3 using TMHMM online tools (http://www.cbs.dtu.dk/services/TMHMM/). The results showed that both MtPIN1 and MtPIN3 consisted of two hydrophobic transmembrane domains and a hydrophilic domain (Supplementary Figure S2).

To characterize their genetic function, the full-length genomic sequences of *MtPIN1* and *MtPIN3* were blasted in the *Medicago truncatula* Mutant Database (medicago-mutant.noble.org) to search the putative *Tnt1* retrotransposon-tagged mutants. Two mutant lines of *MtPIN1* were obtained, and a single *Tnt1* was inserted into the 193 bp of the first exon in *mtpin1-1* and 1516 bp of the second exon in *mtpin1-2*, respectively (Figure 1A). PCR amplification of the *MtPIN1* genomic sequence from wild-type and two mutant lines confirmed that a 5.3 kb *Tnt1* retrotransposon insertion existed in mutants (Figure 1B). RT-PCR was performed to detect if *MtPIN1* was expressed in mutants. The results showed that *Tnt1* insertion blocked normal transcription of *MtPIN1* in *mtpin1-1* and *mtpin1-2* (Figure 1C). Moreover, the *Tnt1* was respectively inserted into 302 bp of the first exon in *mtpin3-1* and 1042 bp of the first exon in *mtpin3-2* (Figure 1D). PCR amplification of *MtPIN3* genomic sequence from wild-type and two mutant lines confirmed that a 5.3 kb *Tnt1* retrotransposon insertion existed in mutants (Figure 1E). *MtPIN3* expression was not detected in *mtpin3-1* and *mtpin3-2* by RT-PCR, indicating that the transcription of *MtPIN3* was disrupted by *Tnt1* insertion (Figure 1F).

### 2.3. MtPIN1 and MtPIN3 Synergistically Regulate the Development of Leaves

**Figure 2:**
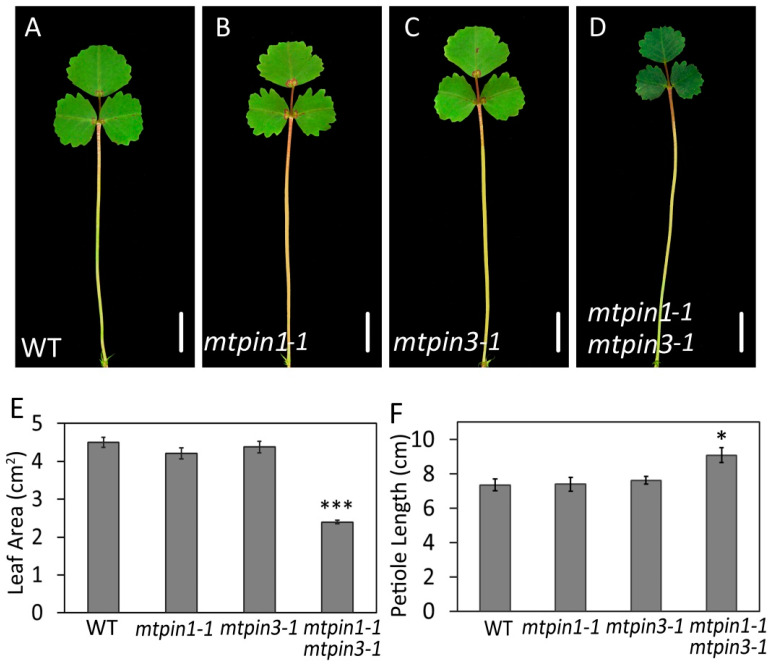
Phenotypes analysis of *mtpin1*, *mtpin3*, and *mtpin1 mtpin3*. (**A**–**D**) Fully expanded leaves from 45-day-old wild-type, *mtpin1-1*, *mtpin3-1*, and *mtpin1-1 mtpin3-1* plants at vegetative stage. (**E**) Measurement of leaf area in wild-type (WT) and mutants. (**F**) Measurement of petiole length in wild-type and mutants. Values are the means ± SD (*n* = 20); * *p* < 0.05, *** *p* < 0.001. Bars = 1 cm.

In *Arabidopsis*, successive mutations of *PIN3*, *PIN4*, and *PIN7* lead to the pleiotropic developmental defects in different organs, such as embryo, roots, and branches [40]. In *M. truncatula*, all of the *mtpin1* and *mtpin3* single mutant alleles did not display obvious defects compared with wild-type (Figure 2A–C; Supplementary Figure S3). To explore whether *MtPIN1* and *MtPIN3* play roles redundantly, *mtpin1 mtpin3* double mutants were generated in two mutant allele combinations. *mtpin1-1*, *mtpin3-1*, and *mtpin1-2 mtpin3-2* displayed the similar developmental defects in leaf and petiole development (Figure 2D; Supplementary Figure S3). Leaf area was significantly decreased, and petiole length was increased in *mtpin1 mtpin3* double mutants, compared with those in wild-type and two single mutants (Figure 2E,F). These observations suggest that *MtPIN1* and *MtPIN3* synergistically regulate the development of leaf size and petiole length in *M. truncatula*.

Previous studies showed that loss of function of *PIN3* in *Arabidopsis* results in short hypocotyls [23]. Successive mutations on *PIN3*,*4*,*7* exhibit striking defects in embryo and root in *Arabidopsis* [22,23]. The orthologous of *PIN1*,*3*,*4*,*7* have been identified in other species. *PttPIN1*–*3* are involved in modulating vascular cambium development in wood-forming tissues [41], *LaPIN1*–*3* act in regulating hypocotyls growth [42], and *BjPIN1*–*3* are expressed in various organs and mainly regulate vascular development [43,44]. These observations indicate that PINsmediated auxin distribution is involved in multiple developmental processes among species.

### 2.4. The Cell Size and Arrangement in Leaves are Altered in mtpin1 mtpin3 Double Mutant

**Figure 3:**
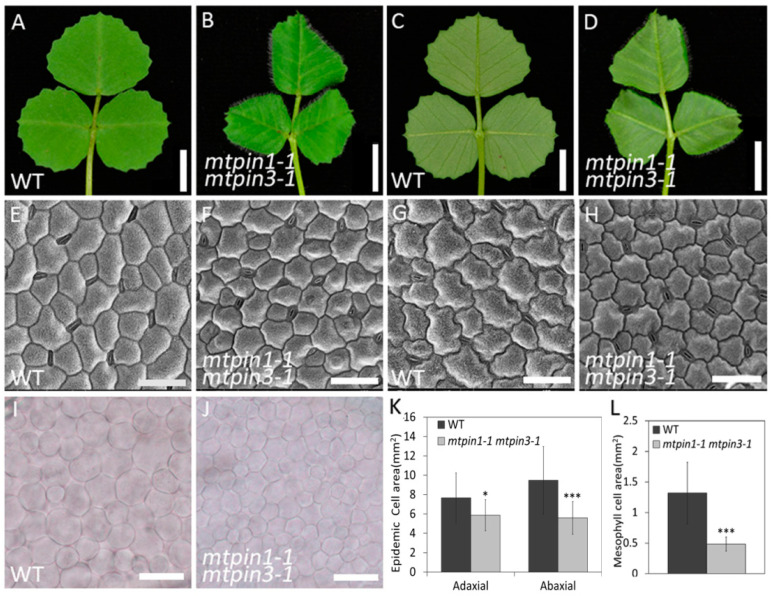
Defects in leaf development in *mtpin1-1 mtpin3-1* double mutant. (**A**,**B**) the adaxial side of a fully expanded leaf in 45-day-old wild-type and *mtpin1-1 mtpin3-1* at the vegetative stage. (**C**,**D**) the abaxial side of a leaf in wild-type and *mtpin1-1 mtpin3-1*. (**E**–**H**) scanning electron microscope (SEM) images of epidermal cells of the adaxial (E and F) and abaxial (**G**,**H**) sides of a fully expanded leaf from 45-day-old wild-type (WT) and *mtpin1-1 mtpin3-1*. (**I**,**J**) Phase contrast microscopy images of mesophyll cells of wild-type and *mtpin1-1 mtpin3-1*. (**K**) Measurement of epidemic cell area of the adaxial and abaxial sides of a leaf of wild-type and *mtpin1-1 mtpin3-1*. (**L**) Measurement of mesophyll cell area of wild-type and *mtpin1-1 mtpin3-1*. Values are the means ± SD (*n* = 50); * *p* < 0.05, *** *p* < 0.001. Bars = 1 cm in (**A**–**D**), 50 um in (**E**–**H**), 20 um in (**I**,**J**).

Compared with the leaves in wild-type (Figure 3A,C), the leaves in *mtpin1-1 mtpin3-1* were curled downwards (Figure 3B,D). To investigate if leaf polarity is altered in double mutants, qRT-PCR was performed to test the expression level of leaf polarityrelated genes, including the *HD-ZIPIII* gene family and *PHAN* gene. The results showed that the expression of those genes did not change significantly (Supplementary Figure S4). To further characterize the defects in *mtpin1-1 mtpin3-1* leaves, scanning electron microscope (SEM) analysis was performed to compare epidermal cells between mutants and wild-type. The observation showed that epidermal cells became relatively round in both the adaxial and abaxial side of *mtpin1-1 mtpin3-1* leaves, compared with those in wild-type (Figure 3E–H). Moreover, the epidermal cells in both sides of leaves in double mutants were smaller than those in wild-type (Figure 3K). Then, the mesophyll cell morphology in wild-type and *mtpin1-1 mtpin3-1* was analyzed by phase contrast microscope (PCM). The results showed that mesophyll cells in double mutants were arranged more compactly (Figure 3I,J), and the mesophyll cell size was also decreased in double mutants (Figure 3L).

### 2.5. Auxin Responsiveness and Free Auxin Content Altered in Mtpin1 Mtpin3

**Figure 4:**
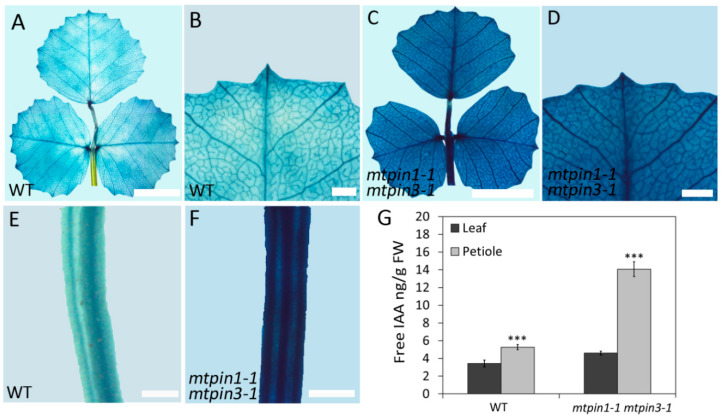
Auxin accumulated in leaf and petiole in *mtpin1-1 mtpin3-1*. (**A**,**B**) *DR5:GUS* expression in adult leaf of wild-type. Close view is shown in (**B**). (**C**,**D**) *DR5:GUS* expression in adult leaf of *mtpin1-1 mtpin3-1*. Close view is shown in (**D**). (**E**,**F**) *DR5:GUS* expression in petiole of wild-type and *mtpin1-1 mtpin3-1*. (**G**) Measurement of free auxin content in leaf and petiole of 45-day-old wild-type (WT) and *mtpin1-1 mtpin3-1*. Values are the means and SD of three biological replicates. *** *p* < 0.001. Bars = 1 cm in (**A**,**C**), 1 mm in (**B**,**D**–**F**).

To test whether *MtPIN1* and *MtPIN3* modulate the development of the lateral organ by affecting auxin distribution, the *DR5* promoter-b-glucuronidase (GUS) marker gene was transferred into *mtpin1-1 mtpin3-1* plants. GUS staining showed that auxin was accumulated in the midvein, marginal serration tips, and pulvinus in wild-type (Figure 4A,B). The expression pattern of *DR5:GUS* was not significantly altered in *mtpin1-1 mtpin3-1*, however, stronger GUS signaling was displayed in the leaf of double mutants (Figure 4C,D). Meanwhile, a pronounced GUS signal was observed in the petiole of *mtpin1-1 mtpin3-1*, compared with that in wild-type (Figure 4E,F). These observations indicate that auxin responsiveness is highly increased in *mtpin1-1 mtpin3-1*, implying that auxin is accumulated in double mutants. To verify this hypothesis, the free IAA content of leaf blade and petiole was measured. The data showed that free IAA content was significantly increased in both leaf blade and petiole in *mtpin1-1 mtpin3-1* (Figure 4G). Taken together, these data indicate that simultaneous disruption of *MtPIN1* and *MtPIN3* leads to the increased auxin level in leaves, suggesting their roles in auxin transportation.

### 2.6. Transcriptomic Profiles of Leaves in Wild-Type and Mutants

**Figure 5:**
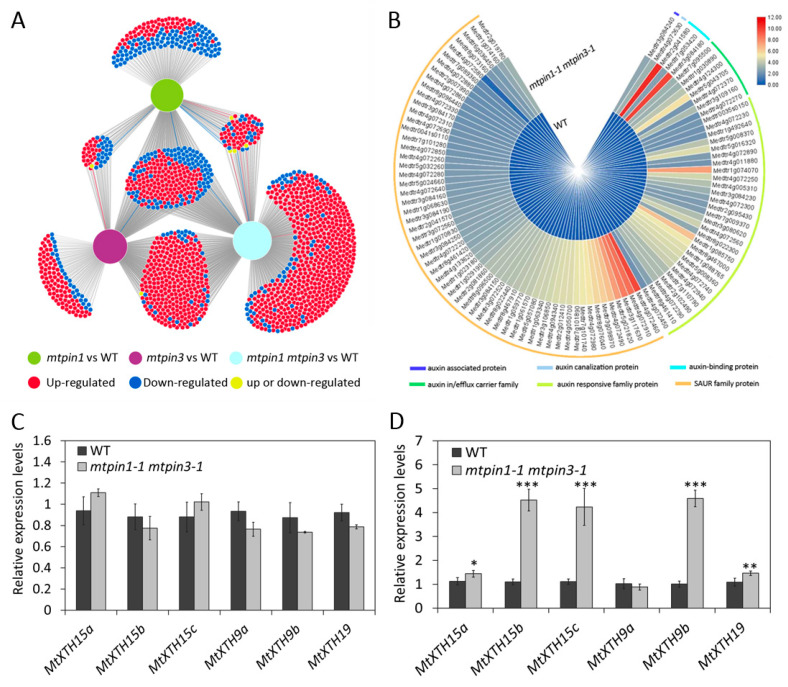
Transcriptomic profiles of wild-type and mutants. (**A**) The full set of DEGs identified in leaves of *mtpin1-1*, *mtpin3-1*, and *mtpin1-1 mtpin3-1*. (**B**) The heatmap of auxin-related DEGs in *mtpin1-1 mtpin3-1*. (**C**,**D**) Relative expression levels of *MtXTH15a*, *MtXTH15b*, *MtXTH15c*, *MtXTH9a*, *MtXTH9b*, and *MtXTH19*, in the leaf (**C**) and petiole (**D**), in wild-type (WT) and *mtpin1-1 mtpin3-1*. Values are the means and SD of three biological replicates. * *p* < 0.05, ** *p* < 0.01, *** *p* < 0.001.

To better understand the involvement of *MtPIN1* and *MtPIN3* in the constitutive shadeavoidance responses, we performed a transcriptomic assay of *mtpin1-1*, *mtpin3-1*, and *mtpin1-1 mtpin3-1*. The leaves were harvested from 45-day-old seedlings and used for transcript profiling analysis by RNA sequencing (RNA-Seq). In total 555, 746, and 1275 differentially expressed genes (DEGs) were identified in *mtpin1-1*, *mtpin3-1*, and *mtpin1-1 mtpin3-1*, respectively (Supplementary Tables S1–S6). The DiVenn tool was used for analyzing the characterizations of DEGs [45]. The results showed that about half of DEGs (614) were identified only in *mtpin1-1 mtpin3-1* (Figure 5A), indicating the functional redundancy between *MtPIN1* and *MtPIN3*. Among those 614 DEGs, 98 DEGs were involved in auxin response and most of them were small auxin response (SAUR) proteins (Figure 5B). These results suggested that auxin response was changed in *mtpin1 mtpin3*. Kyoto Encyclopedia of Genes and Genomes (KEGG) analysis showed that the DEGs in double mutants were involved in multiple developmental processes, including signal transduction, translation, and environmental adaptation (Supplementary Figure S5). Through the KEGG pathways enrichment analysis in *mtpin1 mtpin3*, we found that 96 and 86 DEGs were involved in signal transduction and environmental adaptation. We also carried out enrichment analysis of Gene Ontology (GO) in *mtpin1-1 mtpin3-1*. The results showed that *MtPIN1* and *MtPIN3* participated in multiple biological processes, including metabolic processes, binding, enzyme regulator activity, and response to stimulus (Supplementary Figure S6). Among all enriched GO terms in *mtpin1-1 mtpin3-1’*s DEGs, 67 DEGs were involved in response to stimulus. In general, these results indicated that *MtPIN1* and *MtPIN3* were essential for responding and adapting to environment stimulus. As an environmental stimulus, shade can alter cell wall extendibility by inducing the expression of *XTH* genes, thereby promoting the elongation of petiole to help the plants escape from the shade formed by neighboring plants [46,47]. Since the *mtpin1-1 mtpin3-1* displayed the elongated petiole and downward-curled leaves that are the constitutive shadeavoidance responses phenotype, the transcription of *MtXTH* genes involved in cell elongation were measured. qRT-PCR data showed that the expression level of *MtXTH* genes was essentially unchanged in leaves between wild-type and *mtpin1-1 mtpin3-1* (Figure 5C). This result was consistent with the results of transcriptome analysis, and corresponded to the phenotype of the reduced leaf area and cell size in the double mutant. However, *MtXTH15b*, *MtXTH15c*, and *MtXTH9b* were highly induced in petiole of *mtpin1-1 mtpin3-1*, which corresponded to the phenotype of petiole elongation in the double mutant (Figure 5D). These results imply that the *MtXTH* genes probably respond to the increased auxin level and regulate the cell elongation in the petiole of *mtpin1-1 mtpin3-1*.

### 2.7. Mtpin1 Mtpin3 Exhibit the Constitutive ShadeAvoidance Responses Phenotype under the Normal Growth Condition

**Figure 6:**
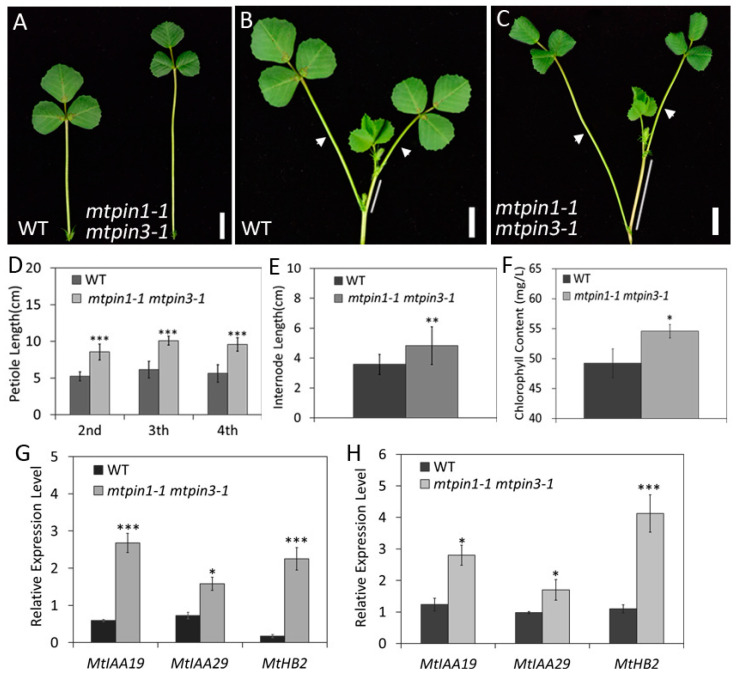
The plants of *mtpin1 mtpin3* exhibit the constitutive shadeavoidance responses. (**A**) Mature compound leaf of 60-day-old wild-type (left) and *mtpin1-1 mtpin3-1* (right). (**B**,**C**) A dissected branch of wild-type (**B**) and *mtpin1-1 mtpin3-1* (**C**). Arrows point to the petioles and lines show the internodes. (**D**) The length of petiole on the different nodes in 60-day-old wild-type (WT) and *mtpin1-1 mtpin3-1*. (**E**) The length of internode in wild-type and *mtpin1-1 mtpin3-1*. (**F**) The chlorophyll content in wild-type and *mtpin1-1 mtpin3-1.* (**G**,**H**) Expression levels of *MtIAA19*, *MtIAA29*, and *MtHB2* at petiole and internode in wild-type and *mtpin1-1 mtpin3-1*. * *p* < 0.05, ** *p* < 0.01, *** *p* < 0.001.

In shade avoidance, shade-tolerant plants compete for light from neighboring plants by increasing hypocotyl growth in young seedlings and increasing stem and petiole growth in older plants, which are beneficial for survival. In *mtpin1-1 mtpin3-1*, the elongated petiole and reduced leaf size are reminiscent of the plant phenotype under the prolonged shade treatment [48,49]. To confirm if the defects in *mtpin1-1 mtpin3-1* were related to shade avoidance response, the petiole development is further investigated in the elder plants (60-day-old). In standard growth condition without shade induction, the length of petioles developed on different nodes of *mtpin1-1 mtpin3-1* was significantly longer than those of wild-type (Figure 6A–D). The internode was also increased in *mtpin1-1 mtpin3-1*, compared with that in wild-type (Figure 6B,C,E). Moreover, the chlorophyll content was increased, which is consistent with the dark green leaves in *mtpin1-1 mtpin3-1* (Figure 6F). In previous reports, *IAA19*, *IAA29*, and *ATHB2* are the marker genes in SAS in *Arabidopsis* [36,50,51,52]. We further examined the transcription of *MtIAA19*, *MtIAA29*, and *MtHB2*. The expression levels of three genes were significantly increased in both petiole and internode of *mtpin1-1 mtpin3-1* (Figure 6G,H). Taken together, these observations indicate that loss of function of *MtPIN1* and *MtPIN3* induces the constitutive shadeavoidance responses phenotype under the normal growth condition.

The elongation of vascular organs in response to light cues can enable plants to escape from the canopy formed by neighboring plants [3,35,53]. The classical shadeavoidance syndrome is characterized by adjustments in plant development in response to a low R:FR ratio perceived by the plant, such as the increased length of hypocotyls, petiole, internode, or stem, and decreased leaf lamina area [31,49]. As found in the previous report, shade can induce the expression of *YUCCA* to initiate de novo auxin synthesis [4]. In *Arabidopsis*, shade and increased free auxin content alter the location of PIN3 to make unidirectional bends of hypocotyl [24], suggesting that local auxin metabolism plays a vital role in shadeavoidance response. In this study, under standard growth condition, *mtpin1 mtpin3* double mutant exhibits elongated petiole and smaller leaf, which is similar to those of plants in the shade. Moreover, increased auxin and chlorophyll content is detected in *mtpin1 mtpin3* double mutant. These defects in *mtpin1 mtpin3* indicate that loss of function in both *MtPIN1* and *MtPIN3* leads to the constitutive shadeavoidance responses phenotype under standard growth condition.

### 2.8. Mtpin1 Mtpin3 Show the Defects in ShadeAvoidance Response in Low R:FR Ratio Light

**Figure 7:**
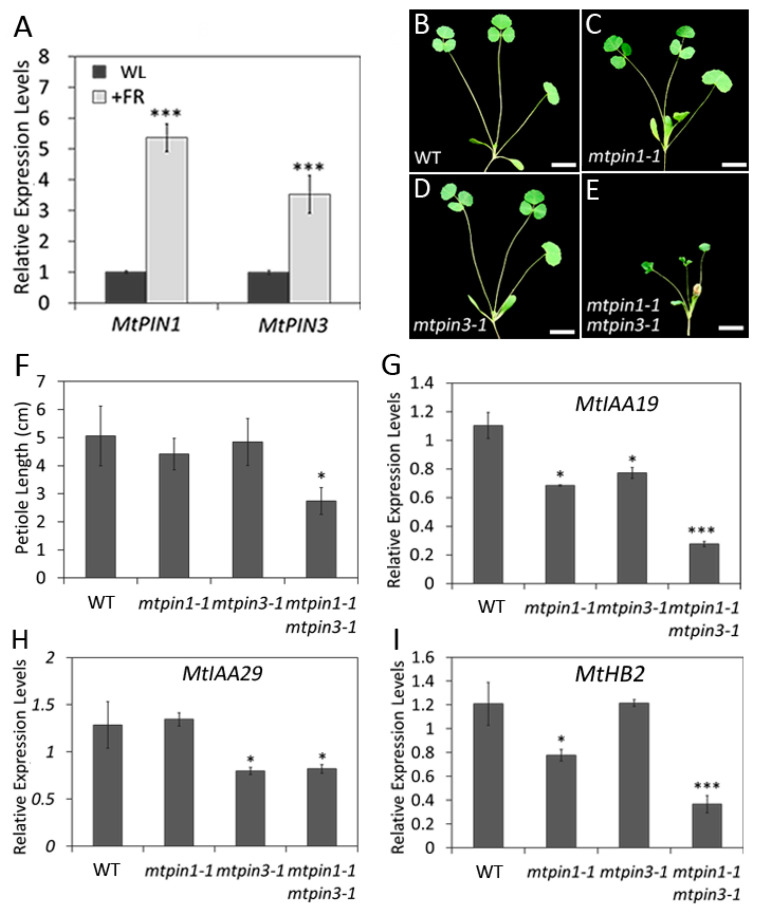
The plants of *mtpin1 mtpin3* fail to respond to low R:FR ratio light. (**A**) Relative expression levels of *MtPIN1* and *MtPIN3* at petiole in wild-type under white light (WL) and a low R:FR ratio light (R:FR ratio = 0.1). (**B**–**E**) Phenotype of 45-day-old wild-type (WT) (**B**), *mtpin1-1* (**C**), *mtpin3-1* (**D**), and *mtpin1-1 mtpin3-1* (**E**) under a low R:FR ratio light. (**F**) The petiole length of wild-type and mutants under low R:FR light. (**G**–**I**) Relative expression levels of *MtIAA19* (**G**), *MtIAA29* (**H**), and *MtHB2* (**I**) under low R:FR light. Values are the means and SD of three biological replicates. * *p* < 0.05, *** *p* < 0.001. Bars = 1 cm in (**B**–**E**).

It has been reported that *PIN3* plays a crucial role in regulating shade-induced hypocotyl elongation by altering auxin distribution in *Arabidopsis* [24]. To check if *MtPIN1* and *MtPIN3* respond to shade, their expressions were measured in wild-type under white light (WL) and low Red:Far-Red (R:FR = 0.1) ratio light. The results showed that *MtPIN1* and *MtPIN3* were upregulated under low R:FR ratio light, indicating that shade is able to induce their expression (Figure 7A). To investigate the roles of *MtPIN1* and *MtPIN3* in shade growth condition, the development of plants was compared under the low R:FR ratio light. The observations showed that the petiole length was significantly decreased and the growth of *mtpin1-1 mtpin3-1* was severally repressed, leading to the dwarf phenotype (Figure 7B–F). These results indicated that *mtpin1-1 mtpin3-1* double mutant fails to respond to low R:FR ratio light. These data suggest that *MtPIN1* and *MtPIN3* are required for the shadeavoidance response in *M. truncatula*. Furthermore, the expressions of *MtIAA19*, *MtIAA29*, and *MtHB2* were examined. The expression levels of all three genes were significantly decreased in double mutant under the low R:FR ratio light, compared with those in wild-type (Figure 7G–I). Taken together, these results indicate that the shadeavoidance response is disrupted in *mtpin1-1 mtpin3-1* double mutant.

In *Arabidopsis*, *PIN3* is expressed in diverse tissues and organs. Loss of function *PIN3* gives rise to multiple developmental defects including failures on phototropism and gravitropism [9,24,25]. Unilateral light and gravity stimulate relocation of *PIN3* to direct auxin flow toward a single side of hypocotyl [25]. Elongation of hypocotyls is one of the strategies for Arabidopsis seedlings to escape from canopy [33,35]. Shade is able to induce the expression of *PIN3* and alter the location of PIN3 in hypocotyls [24]. Free auxin level is also elevated in hypocotyl in low R:FR-exposed wild-type seedlings. However, *pin3-3* mutant failed to elongate hypocotyl and auxin level is not altered in the mutant under the low R:FR ratio light [24]. Hence, *PIN3* plays a vital role in regulating shade-induced auxin accumulation and distribution in hypocotyl. In *M. truncatula*, *mtpin1 mtpin3* displayed the dwarf plants and failed to elongate petiole and internode in shade, indicating that *mtpin1 mtpin3* did not respond to low R:FR ratio light. In addition, the expression levels of marker genes of SAS are not induced in *mtpin1 mtpin3*. Therefore, *PIN3* and its orthologous in *M. truncatula* genes are functionally conserved in regulating shadeavoidance response under the low R:FR ratio light.

## 3. Materials and Methods

### 3.1. Plant Materials and Growth Conditions

The mutants used in this study were in the genetic background of the R108 ecotype. *mtpin1-1*, *mtpin1-2*, *mtpin3-1*, and *mtpin3-2* mutant lines were isolated from a *Tnt1* retrotransposon-tagged mutant collection of *M. truncatula*. The seeds were polished and germinated at 4 °C for 3 days, then transferred into a growth chamber supporting a 16 h day/8 h night photoperiod for 7 days. Then, plants were transferred into different growth conditions, white light and simulated shade (R:FR = 0.1), with the 16 h light/8 h dark photoperiod for 1 month.

### 3.2. Phylogenetic Analysis

Amino acid sequences of *A. thaliana* and *M. truncatula* were downloaded from TAIR (https://www.arabidopsis.org/) and Phytozome (https://phytozome.jgi.doe.gov/), respectively. The alignment of multiple amino acids sequenced was performed using ClustalW (http://www.clustal.org/, v2.1, Dublin, Ireland). The neighbor-joining phylogenetic tree was constructed using the MEGA-X software (http://www.megasoftware.net/, v10.2.2, Tempa, AZ, USA), ~1000 bootstrap replicates were used.

### 3.3. RNA Extraction and Quantitative Real-time PCR (qRT-PCR) Analysis

The adult leaf and petiole tissues of 45-day-old wild-type and mutants were collected for total RNA isolation. The samples were fully grinded. Then, TRIzol-RT Reagent (Invitrogen, Carlsbad, CA, USA) was used for isolating total RNA. RT-PCR and qRT-PCR analysis were performed as described previously [54]. All qRT-PCR primers used are listed in Supplementary Table S7.

### 3.4. Transcriptomic Analysis

For transcriptomic analysis, three biological replicates of leaves were harvested from 45-day-old plants of wild-type, *mtpin1-1*, *mtpin3-1*, and *mtpin1-1 mtpin3-1*. RNA samples were sequenced on a BGISEQ–500 platform at BGI Genomics Institute (BGI–Shenzhen, Shenzhen, China). For each replicate, RNAsequencing generated more than 20 million raw reads. Raw reads were first purified by trimmonmatic (v0.37, BGI-Shenzhen, Shenzhen, China) [55]. Adapter sequences, low-quality reads, and reads containing more than 5% unknown nucleotides were filtered out from raw reads. Then, clean reads were aligned against the annotated *M. truncatula* reference transcriptome using Bowtie (v4.0, Baltimore, MD, USA) [56]. RSEM (RNA-Seq by Expectation Maximization) was used for gene expression analysis [57] and R package DEGseq (Differentially Expressed Gene Identification for RNA-seq data) was used for identifying differentially expressed genes (DEGs) [58]. All DEGs characterized were up/downregulated more than two-fold and a false discovery rate (FDR) < 0.001. The hypergeometric test of *p*-value adjusted by the FDR method was used to evaluate the enrichment of gene ontology (GO) terms and the KEGG pathway.

### 3.5. Scanning Electron Microscopy (SEM) and Phase Contrast Microscopy (PCM)

For scanning electron microscopy analysis, 25% glutaraldehyde was diluted by phosphate buffer (PH = 7.0) into 3% final fixation solution, and then leaf tissue samples were fixed in 3% glutaraldehyde overnight. The tissues were washed and dehydrated with gradient ethanol. Tissues samples were dried and coated with gold and examined under a Quanta 250 FEG scanning electron microscope at an accelerating voltage of 5 kV (FEI, Hillsboro, OS, USA). For phase contrast microscopy analysis, fully expanded adult leaves were detached from wild-type, first fixed in ethanol:acetic acid (6:1) solution for 2 h twice in total at room temperature. The tissue samples were treated by chloraldurate:glycerol:water (8:1:3) solution, and examined by the fluorescence microscope with optical filter (Nikon NI-SS, Tokyo, Japan).

### 3.6. Quantification of Chlorophyll

Fully expanded leaves were collected from wild-type and mutants. The samples were fully grinded and immediately added to 3 mL of 80% acetone containing 1 mM KOH. After centrifugation, supernatants were used for measuring chlorophyll contents by different wavelengths.

### 3.7. β-Glucuronidase Staining and Quantification of Auxin

For GUS staining analysis, leaf and petiole were collected from wild-type and mutants. The GUS activity was histochemically detected as previously described [30]. Mature adult leaves and petioles were detached from 45-day-old plants of wild-type and *mtpin1-1 mtpin3-1* for quantification of free IAA by liquid chromatography-tandem mass spectrometry (LC-MS/MS, 8030 plus, Shimadzu, Tokyo, Japan) as follows: 200 mg of fresh sample frozen by liquid nitrogen was wellhomogenized using a TissueLyser homogenizer (QIAGEN, Hilden, Germany) with a small glass pestle in a 2 mL vial. After 1.0 mL of 80% methanol was added, the homogenates were thoroughly mixed in an ultrasonic bath, and then maintained overnight at 4 °C. After being centrifuged at 15,200× *g* for 10 min, the supernatant was collected and vacuum-dried in a Jouan RCT-60 concentrator. The dried extract was dissolved in 200 µL of sodium phosphate solution (0.1 mol/L, pH 7.8) and then passed through a Sep-Pak C18 Cartridge (Waters, Milford, MA, USA). The cartridge was eluted with 1.5 mL of 80% methanol, and the eluate was again vacuumed to dryness. After dissolving it in 10 mL of 10% methanol, 5 µL of this solution was injected into the LC-MS/MS system. Liquid chromatography was performed using a 2.0 mm I.D. × 75 mm Shim-pack XR-ODS column (2.2 μM, Shimadzu, Tokyo, Japan) at a column temperature of 40 °C. The mobile phase comprising of solvent A (0.02% *v/v* aqueous acetic acid) and solvent B (100% *v/v* methanol) was employed in a gradient mode (time/A concentration/B concentration (min/%/%) for 0/90/10; 5/10/90; 6/10/90; 6.1/80/20) at an eluant flow rate of 0.3 mL/min. The mass system was set to multiple reaction monitoring (MRM) and negative ion mode using electrospray ionization (ESI). The operating conditions such as nebulizing gas flow, drying gas flow, desolvation temperature, and heat block temperature were respectively optimized. Deuteriumlabeled IAA (Olchemim, Olomouc, Czech Republic) was used as an internal standard. Collision energy of –16 eV and mass-to-charge ratio (m/z) of 174.2 were employed [59].

## 4. Conclusions

Taken together, our data suggest that *MtPIN1* and *MtPIN3* synergistically function in shadeavoidance response. Under the normal growth condition, *MtPIN1* and *MtPIN3* repress the constitutive shadeavoidance responses by regulating the auxin distribution in petiole and leaf. Under the low R:FR ratio light, the expression of *MtPIN1* and *MtPIN3* is induced and responds to shade. Therefore, unlike *PIN3* in *Arabidopsis*, *MtPIN1* and *MtPIN3* play dual roles in regulation of shadeavoidance response under different environments. Identification of *PIN3* and its orthologs will help to provide insight into their functional specialization and conservation among species.

## Supplementary Materials

Supplementary materials can be found at https://www.mdpi.com/1422-0067/21/22/8742/s1.

## Abbreviations

CDS	coding region sequences
DEGs	differentially expressed genes
IAA	indole-3-acetic acid
KEGG	Kyoto Encyclopedia of Genes and Genomes
KOH	potassium hydroxide
PAT	polar auxin transportation
PCM	phase contrast microscope
PM	plasma membrane
qRT-PCR	quantitative RT-PCR
R:FR	Red:Far-Red light ratio
SAS	Shadeavoidance syndrome
SEM	scanning electron microscope
SAUR	small auxin response
WL	white light
XTH	xyloglucan endotransglucosylase
