# Supplementary material for: MtPIN1 and MtPIN3 Play Dual Roles in Regulation of Shade Avoidance Response under Different Environments in Medicago truncatula
Source: Int J Mol Sci. 2020 Nov 19;21(22):8742. doi: 10.3390/ijms21228742 (PMC7699406; doi:10.3390/ijms21228742)
Supplement: Supplementary file 1 [file ijms-21-08742-s001.zip › ijms-990938 revised sup/Supplementary File/ijms-Supplementary figures-REVISED-1109.docx]

**Supplemental Figures**





Figure S1. Phylogenetic tree analysis of MtPIN1 and MtPIN3. (A) Phylogenetic tree analysis of PIN family proteins in M. truncatula and A. thaliana. (B) to (C) Relative expression levels of *MtPIN1* and *MtPIN3* in different organs in wild-type. Values are means ± SD of three biological replicates. JL: juvenile leaf; AL: adult leaf; PI: petiole; SB: shoot bud; F: flower; S: stem; P: pod; Sd: seed; R: root.


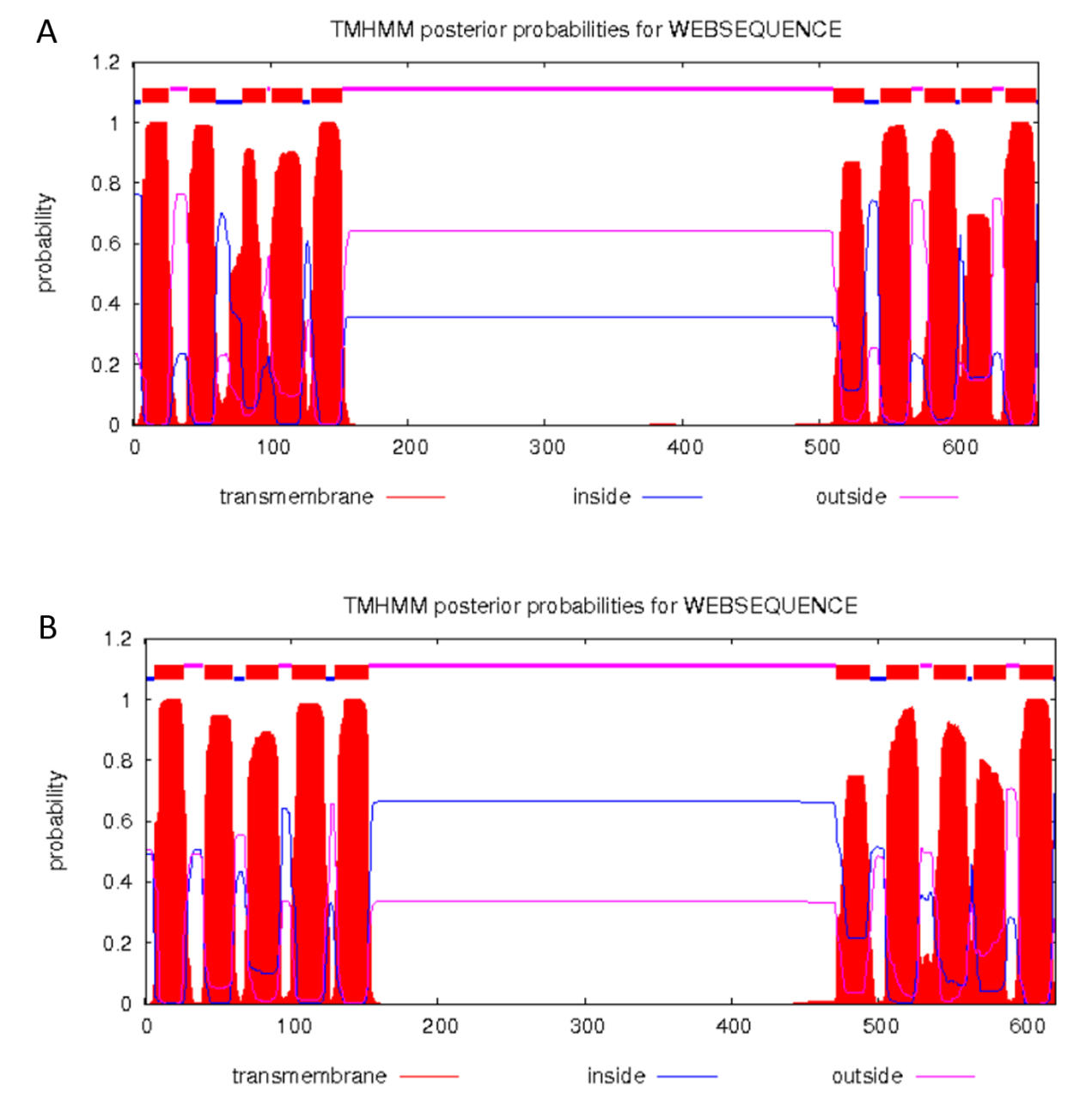


Figure S2. Prediction of transmembrane domain of *MtPIN1* and *MtPIN3*. TMHMM: http://www.cbs.dtu.dk/services/TMHMM/. (A) Prediction of transmembrane domain of MtPIN1. (B) Prediction of transmembrane domain of MtPIN3.


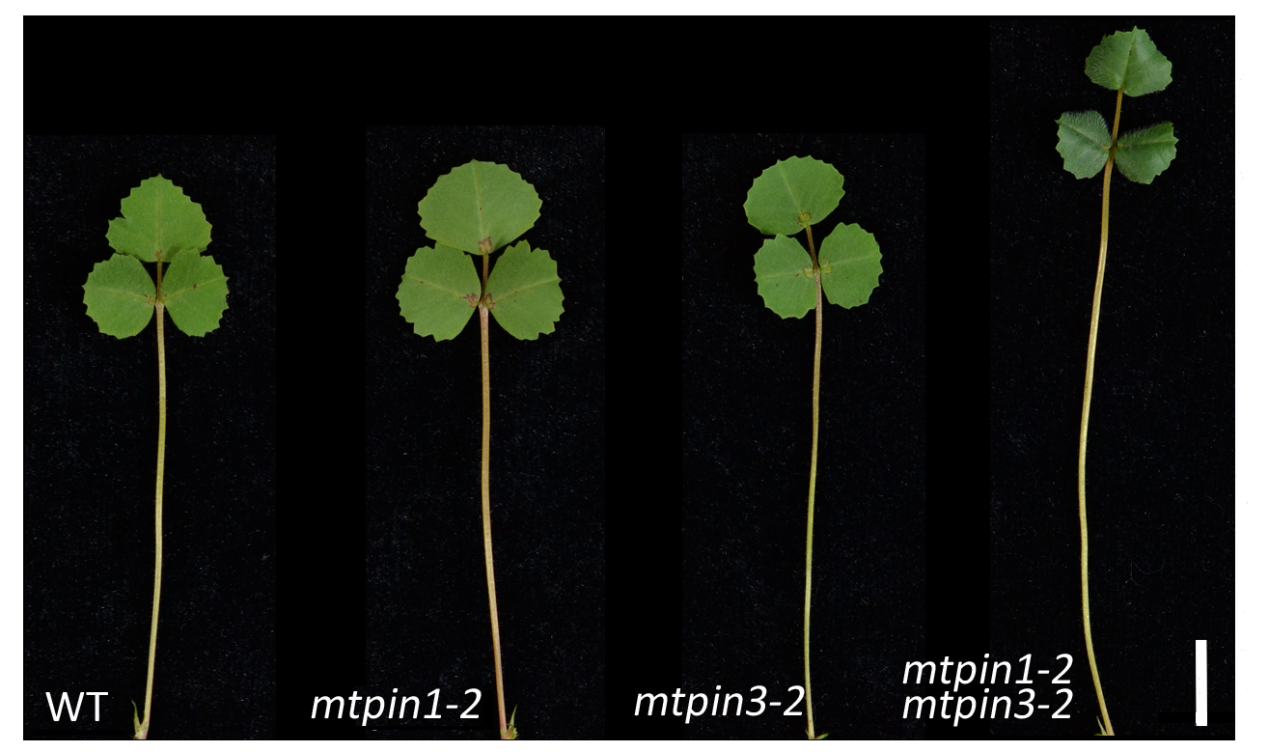


Figure S3. Phenotype of *mtpin1-2*, *mtpin3-2*, *mtpin1-2 mtpin3-2*. Bars = 1 cm.


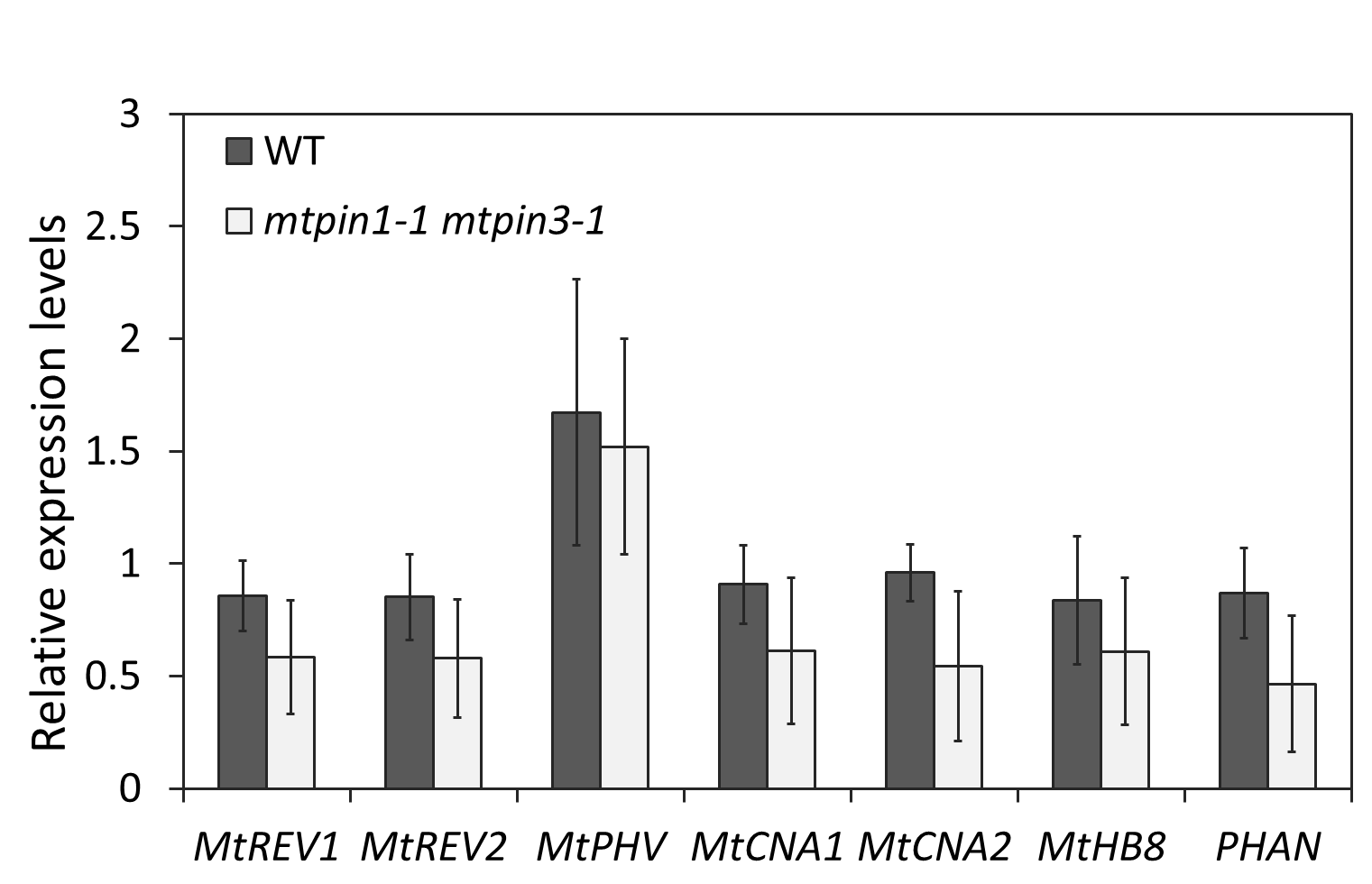


Figure S4. Relative expression levels of genes involved in establishment and maintenance of adaxial polarity of leaf in *M.truncatula.*


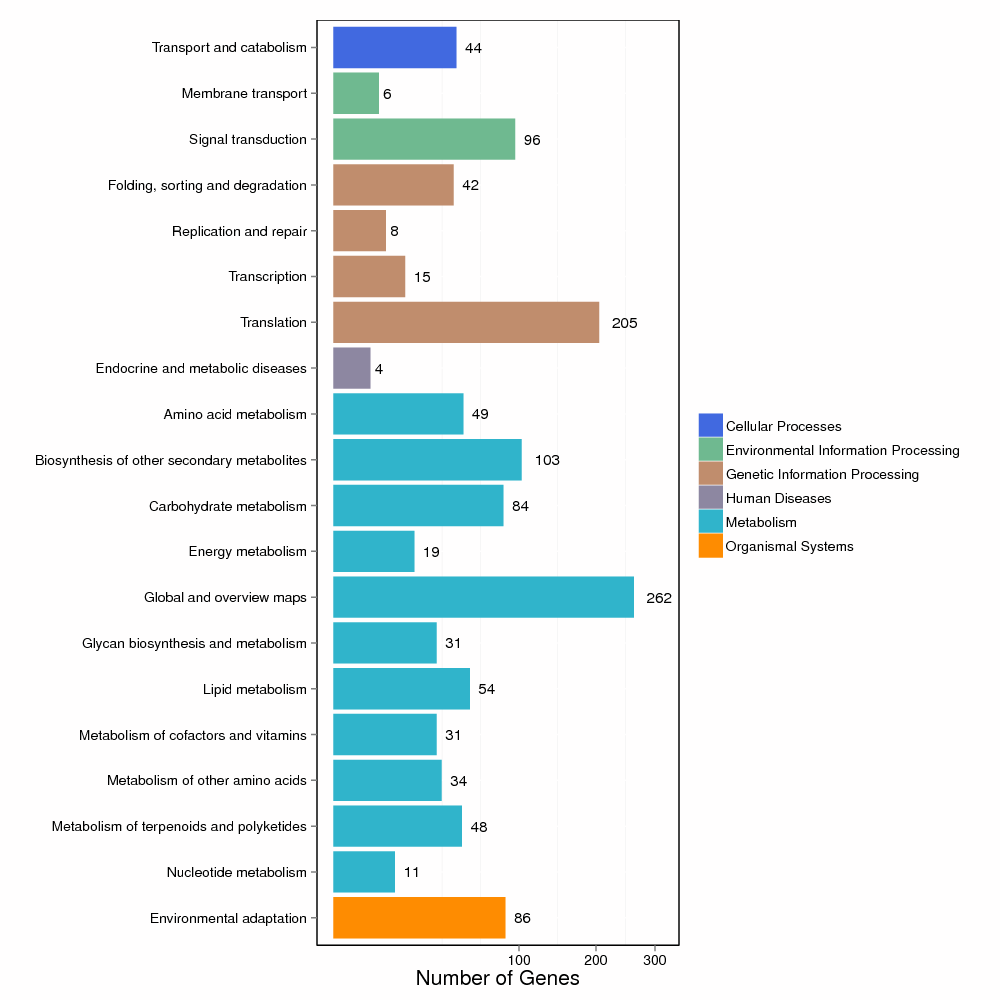


Figure S5. Analyze on KEGG pathways enrichment in *mtpin1-1 mtpin3-1.*


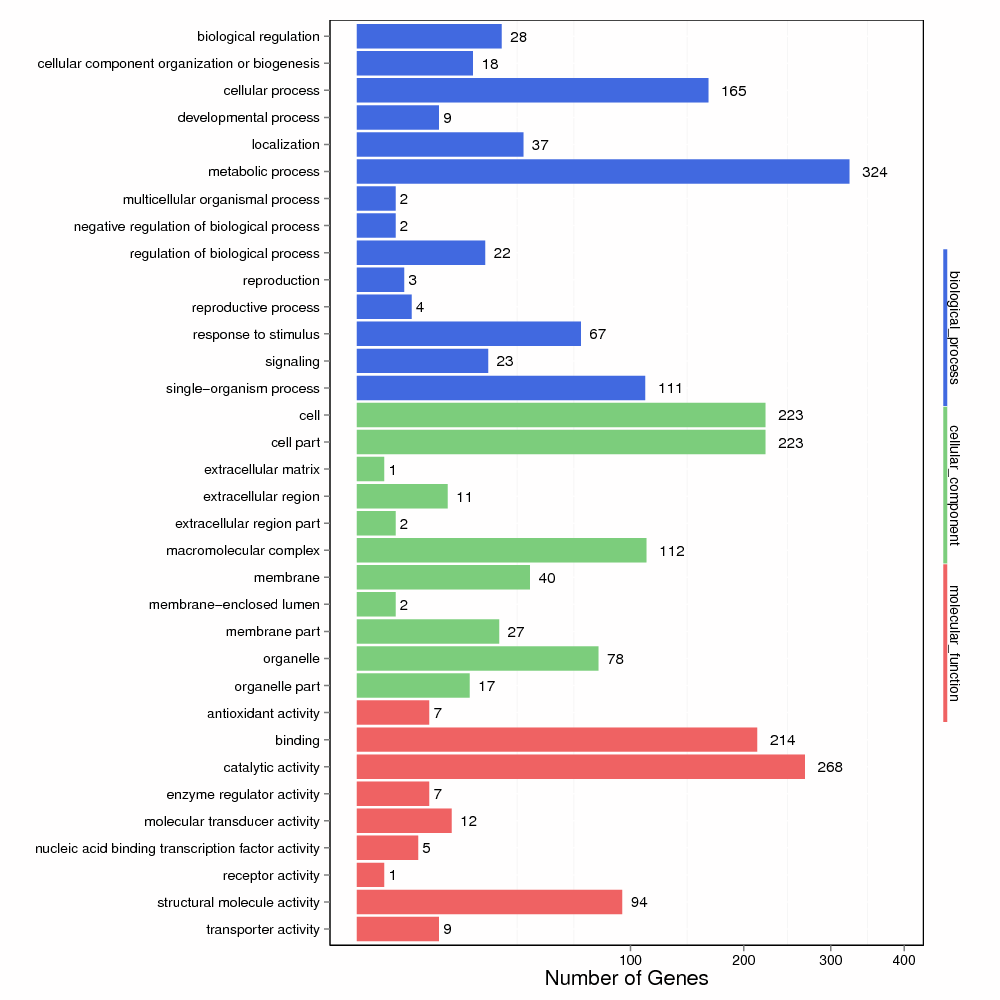


Figure S6. Analyze on GO terms enrichment in *mtpin1-1 mtpin3-1.*
